# The complete mitochondrial genome of *Wellcomia compar* (Spirurina: Oxyuridae) and its genome characterization and phylogenetic analysis

**DOI:** 10.1038/s41598-023-41638-9

**Published:** 2023-09-02

**Authors:** Chunmao Huo, Fengyun Bao, Hong Long, Tingyang Qin, Shibin Zhang

**Affiliations:** Laboratory Animal Center, Zunyi Medical University, Zunyi, 563006 China

**Keywords:** Mitochondrial genome, Animal breeding

## Abstract

*Wellcomia compar* (Spirurina: Oxyuridae) is a pinworm that infects wild and captive porcupines. Despite clear records of its morphological structure, its genetics, systematics, and biology are poorly understood. This study aimed to determine the complete mitochondrial (mt) genome of *W. compar* and reconstruct its phylogenetic relationship with other nematodes. We sequenced the complete mt genome of *W. compar*and conducted phylogenetic analyses using concatenated coding sequences of 12 protein-coding genes (PCGs) by maximum likelihood and Bayesian inference. The complete mt genome is 14,373 bp in size and comprises 36 genes, including 12 protein-coding, two rRNA and 22 tRNA genes. Apart from 28 intergenic regions, one non-coding region and one overlapping region also occur. A comparison of the gene arrangements of Oxyuridomorpha revealed relatively similar features in *W. compar* and *Wellcomia siamensis*. Phylogenetic analysis also showed that *W. compar* and *W. siamensis* formed a sister group. In Oxyuridomorpha the genetic distance between *W. compar* and *W. siamensis* was 0.0805. This study reports, for the first time, the complete *W. compar* mt genome sequence obtained from Chinese porcupines. It provides genetic markers for investigating the taxonomy, population genetics, and phylogenetics of pinworms from different hosts and has implications for the diagnosis, prevention, and control of parasitic diseases in porcupines and other animals.

## Introduction

The Chinese Porcupine (*Hystrix brachyura*) is a member of the rodent family Hystricidae. As a common terrestrial wild animal, it occurs in a wide range of habitats, including primary and secondary forest, cultivated areas and plantations^1^. Artificial breeding of porcupines used to be very popular in China for its high medicinal and ornamental value^2^. Porcupines are relatively well-protected from predators but are subject to a degree of parasitic infestation. Thus, clarifying the species of parasites that porcupines are infected with is important for the control of parasitic diseases. *Wellcomia compar,* the so-called porcupine pinworm that belongs to Oxyuridae nematode, was first reported and the detail of its morphological structure was recorded by Leidy^3^. Similar to that of other pinworms, the life cycle of *W. compar* is simple and typically entails the ingestion of infective eggs orally, and then the larvae infiltrating the glandular fossa of the cecum mature into adult worms. The pinworm can adversely impact porcupine hosts by causing indigestion, weight loss, and poor coat condition. However, so far, few studies have reported the genetics, systematics, and biology of this pinworm.

The phylogeny and classification of nematodes have remained subjects of debate, and the poor resolution conferred by morphologic characteristics has resulted in incongruent classification systems. 18S rRNA was the first genetic marker used for phylogenetic classification of nematodes^4^. This and subsequent analyses based on 18S rRNA have led to the recognition of major lineages of nematodes^5–7^. Recent molecular analyses of mt genome sequences have provided selectable hypotheses for the phylogenetic relationships of nematodes based on 18S rRNA.

Mitochondria (mt) synthesise their own proteins and are involved in various physiological functions^8^. After a long period of evolution, the genetic composition of mt in most metazoans is now present consistently, suggesting that mt genes are important for maintaining the basic functions of mt^9^. The mt genome accumulates mutation rates at a faster rate than the nuclear genome and is characterised by fast evolution, maternal inheritance, and a simple structure. Therefore, it is considered a reliable marker in cladistic systematics^10,11^ and has been widely employed as a genetic marker for identifying and distinguishing organisms, particularly for population genetics among nematode species^12–14^. The 12 protein-coding genes (PCGs) of the mt genome are also often used to elucidate the phylogenetic relationships between species^15–21^.

Despite the availability of numerous advanced sequencing and bioinformatic methods, the 18S rRNA gene remains the primary genetic marker used for phylogenetic construction and molecular identification of Oxyuridomorpha. Additionally, other genes such as Cox1, ITS1, ITS2, and 28S rRNA are also employed for this purpose^22–26^. However, the use of complete genome mt for constructing and characterising Oxyuridomorpha phylogenies is infrequent. To date, only six Oxyuridomorpha species have had their complete mt genomes published^17–21^. Therefore, more mt genomes of Oxyuridomorpha nematodes are needed to provide useful molecular markers for population genetic and phylogenetic studies of pinworms.

In this study, we aimed to sequence and analyse the complete mt genome of *W. compar* and compared it to the gene arrangement order of other pinworms. In addition, we used the concatenated coding sequences (CDS) of 12 PCGs from 33 Spirurina nematodes and an Enoplea nematode to reconstruct a more reliable phylogeny.

## Methods

### Ethics statement

No animal was harmed during the course of this research. This study was approved by the Animal Ethics Committee of the Zunyi Medical University (approval no. LVRIAEC [2016] 2-059).

### Parasite isolates and DNA extraction

Adult *W. compar* parasites were collected from infected porcupines in Zunyi, Guizhou Province, China. The samples were washed with sterile saline, preserved in 100% ethanol and stored in a − 20 °C refrigerator until use. The isolation of total genomic DNA from the samples was carried out using the Wizard Genomic DNA Purification Kit (Promega, Madison, Wisconsin, USA), following the instructions provided by the manufacturer. The DNA concentration and quality of the extracted samples were assessed using a Nanodrop 2000 spectrophotometer. The total DNA was detected by agarose gel electrophoresis using an Agilent 2100 Bioanalyzer.

### Mitochondrial genome sequencing and assembly

The whole genome shotgun strategy was used to construct libraries containing DNA fragments of 400–500 bp that were randomly interrupted by a covaris ultrasonic crusher. Paired-end genomic library (approximately 400 bp inserts) was constructed according to the manufacturer’s instructions (Illumina), and were sequenced by next-generation sequencing technology based on the Illumina HiSeq sequencing platform. A5-miseq v20150522^27^ and SPAdesv3.9.0 (http://cab.spbu.ru/software/spades/)^28^ were used to assemble high-quality second-generation sequencing data to construct contig and scaffold sequences. The contig was compared with the nt library on NCBI using BLAST v2.2.31+, and the contig from mt was screened. Then, collinearity analysis was carried out using the mummer v3.1^29^ software to determine the positional relationship and to fill the gaps between contigs. Finally, A5-miseq software was selected for splicing results, and Pilon v1.18^30^ software was used to correct the results to obtain the final mt sequence.

### Gene annotation and analysis

The complete mt genome sequence was uploaded to the MITOS web server (http://mitos.bioinf.uni-leipzig.de/) for functional annotation^31^. The genetic code was set to 05 invertebrate, and the remaining were set according to the default parameters set by MITOS. For an undetectable tRNA gene, the approximate position was determined by comparison with the corresponding tRNA sequence in the reference genome^32^. The secondary structure was manually drawn to determine the final sequence and structure. The base compositions (%) of the complete mt genome, PCGs, and rRNA genes were calculated using Mega X^33^. The CG View visualisation software was used to map the entire mt genome^34^. The figure of Gene rearrangement was drawn using PhyloSuite v1.2.2 (http://phylosuite.jushengwu.com/installation_packages/PhyloSuite_v1.2.2_Win64_with_plugins.rar)^35^.

### Phylogenetic analysis and genetic distance

The 33 mt genomes of nematode parasites were downloaded from NCBI (supplementary material Table S1), including 32 of Spirurina nematodes (mostly published papers) as ingroups and one of an Enoplea nematode, *Trichuris suis*, as an outgroup. Phylogenetic relationships among the 33 representative nematode species sequences and that of the *W. compar* mt DNA obtained in this study were reconstructed based on the CDS of 12 PCGs excluding the stop codon, and were concatenated. 12 PCGs were aligned in batches with MAFFT^36^ using the '–auto' strategy and codon alignment mode. Each gene was translated into amino acid sequences using the invertebrate mt genetic code in MAFFT and aligned based on its amino acid sequence using the default settings. The alignments were back translated into the corresponding nucleotide sequences, and refined using the codon-aware program MACSE v. 2.03^37^ with the invertebrate mt genetic code, which preserves the reading frame and allows the incorporation of sequencing errors or sequences with frameshifts. Ambiguously aligned fragments of 12 alignments were removed in batches using Gblocks^38^ with the following parameter settings: minimum number of sequences for a conserved/flank position (16/16), maximum number of contiguous non-conserved positions (8), minimum block length (10), and allowed gap positions (with half). The final sequences of the 12 PCGs were concatenated into single alignments for phylogenetic analyses using phylosuite^35^. ModelFinder^39^ was used to select the best-fit partition model (edge-linked) using Bayesian information criterion (BIC). Best-fit partition model for BI and ML trees are listed (supplementary material Tables S2, S3) according to BIC. Bayesian inference phylogenies were inferred using MrBayes v3.2.7a^40^ under the partition model (2 parallel runs, 4 independent Markov chains run for 3,000,000 metropolis-coupled MCMC generations, sampling a tree every 100 generations), in which the initial 25% of sampled data were discarded as burn-in. ML phylogeny was inferred using IQ-TREE v1.6.12 using an edge-linked partition model for 50,000 ultrafast bootstraps^41,42^. Phylograms were drawn using Interactive Tree Of Life (iTOL) v6 (https://itol.embl.de/itol.cgi)^43^. The calculation of pairwise genetic distances between phylogenetic trees was conducted using MEGA X^33^. Variance estimation was performed utilising concatenated PCGs sequences, employing the Bootstrao method with 1000 generations, and modeling the distances using the P-distance.

### Ethics approval and consent to participate

No animal was harmed during the course of this research. We confirm that all experiments were performed in accordance with relevant guidelines and regulations. All experimental procedures involving animals were reviewed, approved and supervised by the Animal Ethics Committee of Zunyi Medical University (approval no. LVRIAEC [2016] 2-059).

## Results and discussion

### Genome structure, organisation and composition

**Figure 1:**
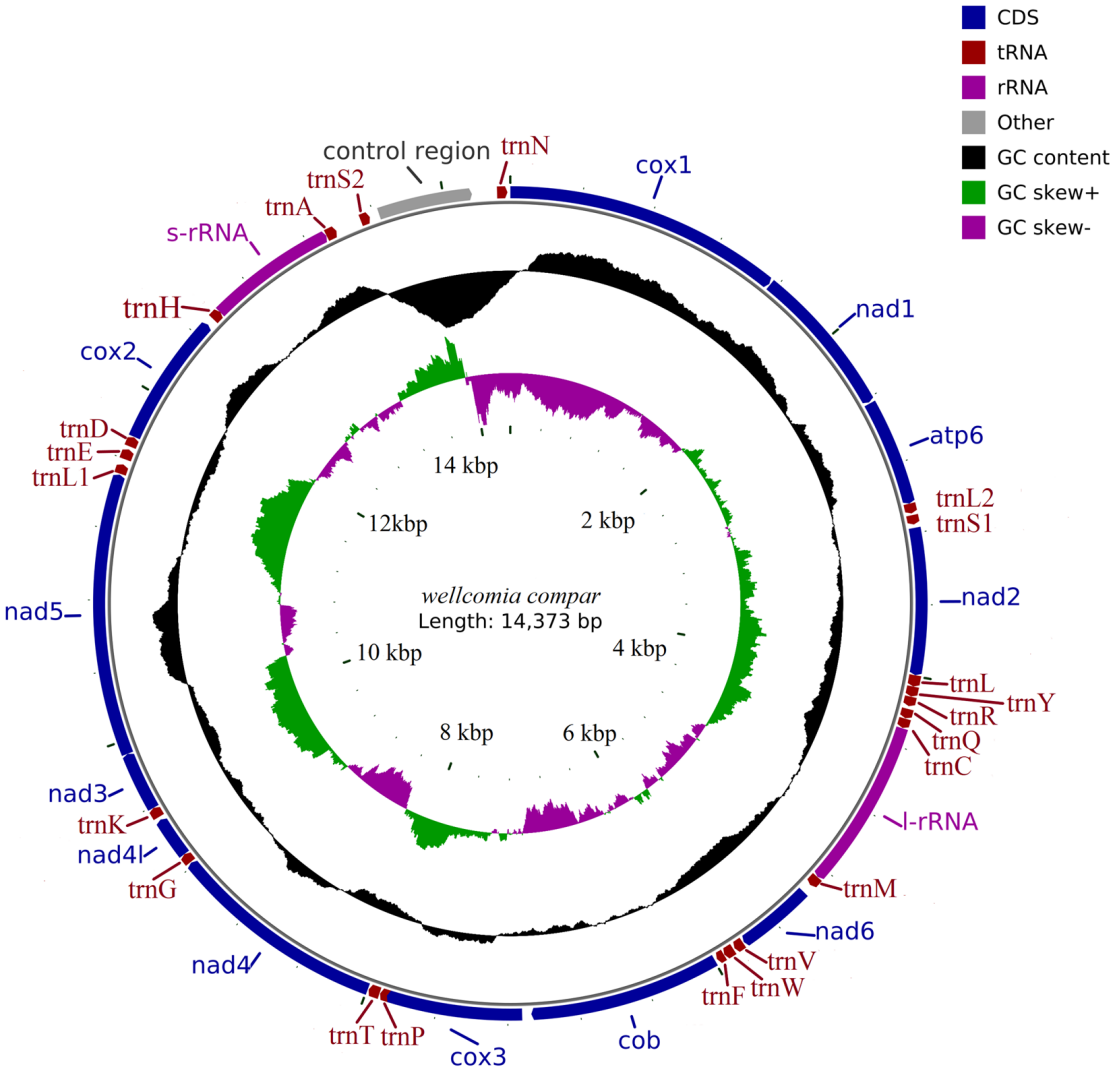
Circular map of the mt genome of *W. compar*. Scale is approximate. 12PCGs and 2 rRNA have standard nomenclature. The 22 tRNA genes are designated by a single-letter code for the corresponding amino acid, with numbers used to distinguish the two leucine and serine designated tRNAs. Grey region rerensents NCR. From the inside to the outside of the circle diagram, the first circle represents the scale, the second circle represents G-C skew, the third circle represents GC content, and the fourth circle represents the arrangement of PCGs, tRNA genes, and rRNA genes on the genome.

The complete mt genome of *W. compar* (GenBank accession No. MW059037) was a 14,373 bp closed-circular molecule (Fig. 1), encoding an entire set of 36 genes, which included 12 PCGs (cox1-3, nad1-6, nad4l, atp6, and cytb), 22 tRNA genes, two rRNA genes (s-rRNA and l-rRNA), and a non-coding region (NCR)) (Table 1). However, this was different from the Enoplea nematode (such as *Trichuris_suis*) gene set, which has an atp8 gene^44^. All genes were transcribed in the same direction on the N-strand. The distribution of genes in the mt genome was identical to those of *Wellcomia siamensis*^17^ and *Syphacia obvelata*^18^. The overall base composition of the mt genome of *W*. *compar* was as follows A: 25.7%; T: 53.0%; G: 16.9%; C: 4.4%; G + C: 21.3%; and A + T: 78.7%. The overall A-T and G-C skews values were -0.346 and 0.586, respectively (Table 2). The intergenic spacer sequence was 613 bp in total and included 28 regions ranging in size from 1 to 147 bp. The overlapping nucleotide fragments were scattered in one place, located between trn H and rrn S (-2 bp).

**Table 1:** Characterisation of the mt genome of *W. compar*.

Gene	Strand	Position	Size(bp)	Initiation codon	Stop codon	Intergenic nucleotide
cox1	N	1–1558	1558	ATA	T(AA)	6
nad1	N	1565–2425	861	ATT	TAG	7
atp6	N	2433–3033	601	TTG	T(AA)	
trnL2	N	3034–3089	56			10
trnS1	N	3100–3152	53			19
nad2	N	3172–3994	823	TTG	T(AA)	
trnI	N	3995–4058	64			
trnY	N	4059–4115	57			1
trnR	N	4117–4170	54			16
trnQ	N	4187–4240	54			2
trnC	N	4243–4296	54			2
rrnL	N	4299–5257	959			1
trnM	N	5259–5318	60			53
nad6	N	5372–5806	435	TTG	TAA	6
trnV	N	5813–5868	56			8
trnW	N	5877–5935	59			2
trnF	N	5938–5994	57			2
cytb	N	5997–7070	1074	TTG	TAG	51
cox3	N	7122–7866	745	TTG	T(AA)	
trnP	N	7867–7924	58			3
trnT	N	7928–7991	64			5
nad4	N	7997–9220	1224	ATT	TAA	4
trnG	N	9225–9280	56			
nad4l	N	9281–9514	234	TTG	TAA	17
trnK	N	9532–9590	59			
nad3	N	9591–9924	334	TTG	T(AA)	
nad5	N	9925–11,508	1584	TTG	TAG	5
trnL1	N	11,514–11,568	55			30
trnE	N	11,599–11,657	59			17
trnD	N	11,675–11,730	56			3
cox2	N	11,734–12,525	792	ATT	T(AA)	1
trnH	N	12,527–12,582	56			− 2
rrnS	N	12,581–13,312	732			1
trnA	N	13,314–13,374	61			147
trnS2	N	13,522–13,575	54			54
NCR	N	13,630–14,159	530			140
trnN	N	14,300–14,357	58

**Table 2:** Base composition of the complete mt genome, PCGs, and rRNA genes of *W. compar*.

Region	A%	C%	G%	T(U)%	A + T%	G + C%	A-T skew	G-C skew
Whole genome	25.7	4.4	16.9	53	78.7	21.3	− 0.346	0.586
PCGs	21.7	4.8	17.8	55.8	77.5	22.6	− 0.440	0.576
atp6	18.6	4.7	19.8	56.9	75.5	24.5	− 0.507	0.619
cox1	22.3	7.5	19.5	50.6	72.9	27.0	− 0.388	0.444
cox2	27.9	4.7	18.6	48.9	76.8	23.3	− 0.273	0.598
cox3	18.4	4.2	18.9	58.5	76.9	23.1	− 0.522	0.640
cytb	24.4	5.0	14.6	56.0	80.4	19.6	− 0.393	0.488
nad1	19.3	5.7	20.2	54.8	74.1	25.9	− 0.480	0.561
nad2	23.7	2.7	15.7	58.0	81.7	18.4	− 0.420	0.709
nad3	21.3	1.8	16.5	60.5	81.8	18.3	− 0.480	0.803
nad4	20.8	4.8	16.2	58.2	79.0	21.0	− 0.473	0.541
nad4l	20.1	2.6	18.4	59.0	79.1	21.0	− 0.492	0.755
nad5	20.1	4.2	19.1	56.6	76.7	23.3	− 0.477	0.642
nad6	21.1	3.4	12.0	63.4	84.5	15.4	− 0.500	0.552
l-rRNA	31.7	3.4	15.6	49.2	80.9	19.0	− 0.216	0.639
s-rRNA	34.2	5.3	18.2	42.3	76.5	23.5	− 0.107	0.547

### Gene annotation and analysis

The mt genome of *W. compar* encodes 12 PCGs, which contain 3420 codons with a total length of 10,260 bp. The content of A + T was 77.5%, which was far higher than G + C. The nucleotides in metazoan mt genomes are not randomly distributed, and this nucleotide bias is often linked to the unequal usage of codons^45^. The nucleotide usage of the 12 PCGs in the mt genome of *W. compar* is shown in Table 3. The codons TTT (phenylalanine, 19.2%), TTA (leucine-2, 8.2%), ATT (isoleucine, 7.5%) usage were most frequent. Therefore, the nucleotides in PCGs were biased towards A and T. Most of the PCGs had the start codon TTG, except for cox 1 that uses ATA, and the other three genes (nad 1, nad 4 and cox 2) that use ATT (Table 1). Three types of stop codons were used: TAG (nad 1, cob and nad 5), TAA (nad 6, nad 4 and nad 4l) and an abbreviated stop codon T (cox 1, atp 6, nad 2, cox 3, nad 3 and cox 2), which is an incomplete TAA stop codon and is completed by the addition of 3` A residues to the mRNA (Table 1).

**Table 3:** Nucleotide codon usage of the 12 PCGs of the mt genome of *W. compar*.

Codon	Count	Codon	Count	Codon	Count	Codon	Count
TTT(F)	657	TCT(S2)	100	TAT(Y)	170	TGT(C)	80
TTC(F)	0	TCC(S2)	0	TAC(Y)	1	TGC(C)	0
TTA(L2)	281	TCA(S2)	11	TAA(*)	3	TGA(W)	51
TTG(L2)	220	TCG(S2)	4	TAG(*)	3	TGG(W)	21
CTT(L1)	10	CCT(P)	54	CAT(H)	51	CGT(R)	35
CTC(L1)	0	CCC(P)	0	CAC(H)	0	CGC(R)	0
CTA(L1)	1	CCA(P)	3	CAA(Q)	19	CGA(R)	0
CTG(L1)	0	CCG(P)	3	CAG(Q)	17	CGG(R)	1
ATT(I)	255	ACT(T)	51	AAT(N)	120	AGT(S1)	121
ATC(I)	0	ACT(T)	0	AAC(N)	1	AGC(S1)	1
ATA(M)	97	ACA(T)	5	AAA(K)	50	AGA(S1)	57
ATG(M)	68	ACG(T)	3	AAG(K)	37	AGG(S1)	20
GTT(V)	243	GCT(A)	48	GAT(D)	70	GGT(G)	139
GTC(V)	2	GCC(A)	1	GAC(D)	0	GGC(G)	0
GTA(V)	45	GCA(A)	3	GAA(E)	46	GGA(G)	54
GTG(V)	38	GCG(A)	4	GAG(E)	23	GGG(G)	21

(F, L2, L1, I, M, V, S1, P, T, A, Y, H, Q, N, K, D, E, C, W, R, S2, G), The letters in parentheses are single-letter abbreviations for 22 amino acids; *, stop codon.

*W. compar* mt DNA contains 22 tRNA genes, which range from 53 bp (trnS1) to 64 bp (trnI and trnT). The two rRNA genes, rrnL and rrnS, were 959 bp and 732 bp in size, respectively (Table 1). rrnL is located between trnC and trnM, and rrnS is situated between trnH and trnA. The A + T content of rrnL and rrnS was 80.9% and 76.5%, respectively (Table 2).

The NCR, located between trnS2 and trnN, was 530 bp in length with a higher A + T content (97.2%) than of any other region of the mt genome.

### Gene rearrangement in Oxyuridomorpha

The Oxyuridomorpha infraorder comprises seven families: Thelastomatoidae, Travassosinematidae, Hystrignathidae, Protrelloididae, Oxyuridae, Pharyngodonidae, and Heteroxynematidae^5^. To date, the mt genomes of many Oxyuridomorpha nematode infraorder lineages are still underrepresented or not represented, except for those of Oxyuridae and Heteroxynematidae. Oxyuridae includes seven species (including *W. compar*) and Heteroxynematidae, and we thus compared the gene rearrangement between the seven species of Oxyuridomorpha.

**Figure 2:**
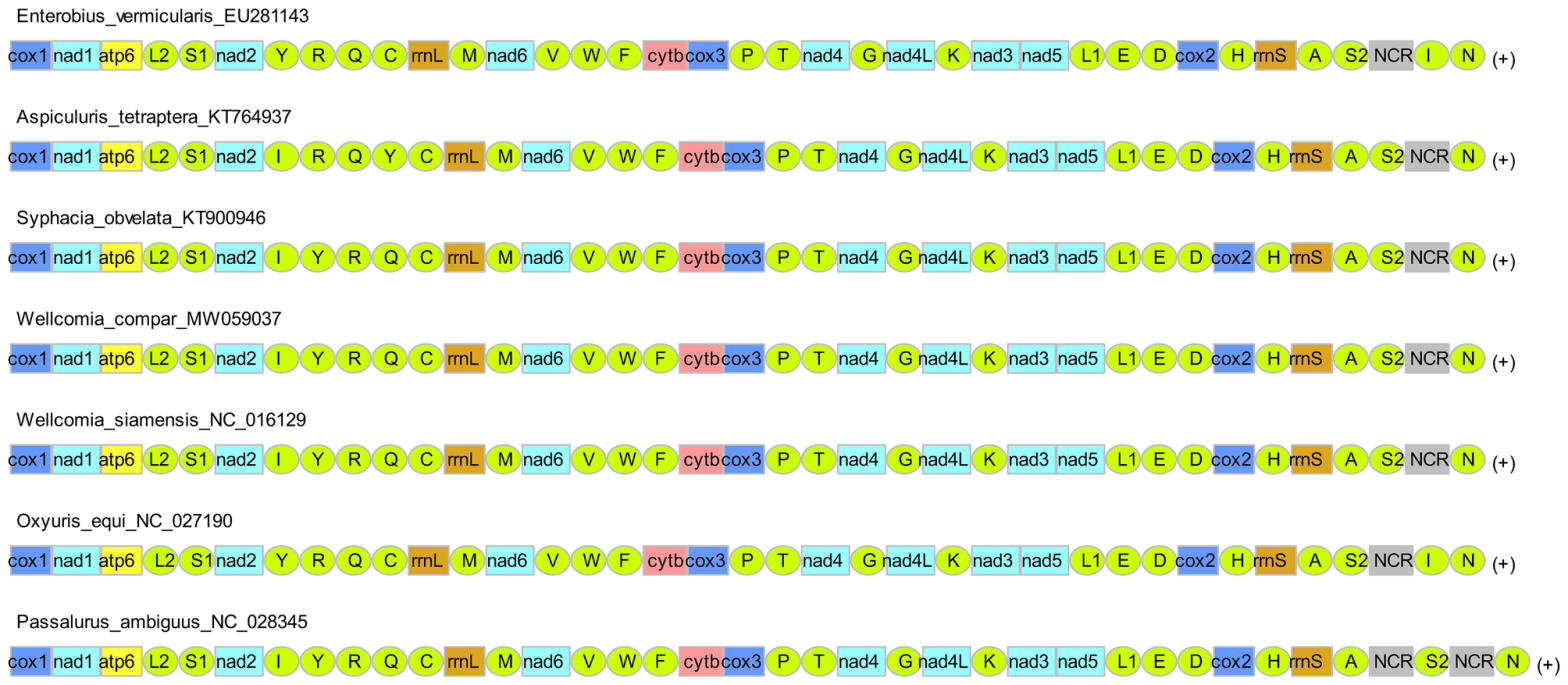
Gene orders in the seven species in Oxyuridomorpha. All genes are transcribed in the same direction on the N-strand.

Gene rearrangement is mainly caused by mutations in the mt^46^, and for which the TDRL model is possibly the most widely accepted explanation hypothesis^47,48^ The mt gene arrangements in the seven species were not the same (Fig. 2), with *W. compar* being consistent with *W. siamensis* and *S. obvelata* but different from *Oxyuris equi*, *Enterobius vermicularis*, *Aspiculuris tetraptera*, and *Passalurus ambiguus*. The main difference was the occurrence of a transposition event (a position change of trnI for *O. equi* and *E. vermicularis*; a position change of trnY for *A. tetraptera*) or the number of NCR. trnI was inserted between NCR and trnN, and trnY was inserted between trnQ and trnC. *P. ambiguus* has two NCRs, consistent with other nematodes^44,49–51^, and an extra NCR in *P. ambiguus* was found between trnA and trnS2. Based on the order of gene arrangement, it can be inferred that *W. compare* has a closer evolutionary relationship with *W. siamensis* and *S. obvelata*.

### Phylogenetic analyses and genetic distance

**Figure 3:**
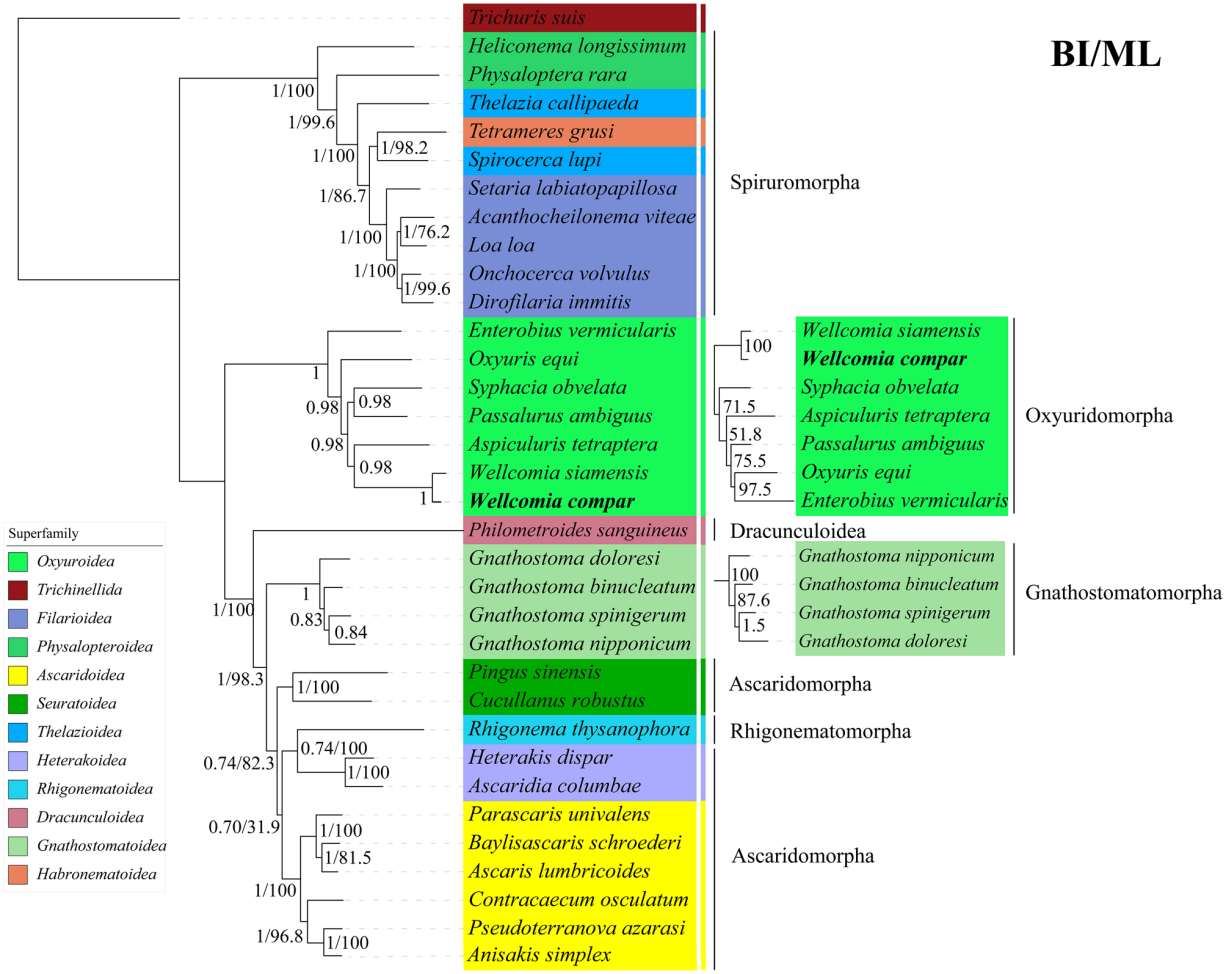
A phylogenetic tree of Spirurina inferred by BI and ML using *Trichuris suis* as the outgroup. Bayesian posterior probability (left) and bootstrap support (right) values of each clade are displayed. *Wellcomia compar* has been bolded. Inconsistent branches in ML were listed separately(right).

Pairwise genetic distances in the phylogenetic tree are displayed in Data S1 of the Supplementary Material, where we find that the genetic distances between species are smaller within the same superfamily than the distances with species from other families. In Oxyuridomorpha, the genetic distance between *W. compar* and *W. siamensis* was 0.0805, which was lower than that between *W. compar* and other pinworms. Phylogenetic analyses of *W. compar* and the selected Spirurina nematodes were performed using maximum likelihood (ML) and Bayesian inference (BI) methods based on concatenated mitochondrial CDS of 12 PCGs (BI/ML [Fig. 3]). The mt genome sequences may provide reliable genetic markers for examining the taxonomic status of nematodes, particularly when PCG sequences are used as markers for comparative analyses^15–21^. Because some superfamilies were represented by a single species, this topology should be interpreted with caution. Phylogenetic analysis showed that the BI and ML trees both divided Spirurina into two clades: Spiruromorpha formed one separate clade, and Oxyuridomorpha + Rhigonematomorpha + Gnathostomatomorpha + Dracunculoidea + Ascaridomorpha formed another clade. The second clade was further sub-divided into two clades, Rhigonematomorpha + Gnathostomatomorpha + Dracunculoidea + Ascaridomorpha and Oxyuridomorpha. These results are consistent with a recent study in Spirurina^47^.

Within Spiruromorpha, many nodes were well-supported. Of note, our results indicate that *Tetrameres grusi* is a sister to *Spirocerca lupi*. Although *S. lupi* and *Thelazia callipaeda* belong to the superfamily Thelazioidea, *T. callipaeda* did not cluster together with *S. lupi*, but instead showed an early diverging position to *S. lupi*. These results are identical to those previously reported^50,52^. Within Ascaridomorpha, the superfamily Ascaridoidea forms a well-supported clade, whereas Rhigonematoidea and Heterakoidea form a sister branch^53,54^, and they are the sister group of Seuratoidea. Notably, Ascaridoidea, Heterakoidea, and Seuratoidea belong to the common infraorder: Ascaridomorpha. The topology of this clade indicated that Rhigonematoidea is closely related to the superfamily Ascaridomorpha. In many early morphology-based classification systems, Rhigonematomorpha was considered to be related to Oxyuridomorpha (pinworms)^55^. Subsequent research based on 18S rRNA phylogeny separated Rhigonematomorpha from Oxyuridomorpha as a distinct group^6^. Our results are inconsistent with those of previous research, and we suggest that it may belong to Ascaridomorpha.

Our results show that Dracunculoidea is more closely related to Ascaridomorpha than Spiruromorpha, which is in agreement with recent studies^42^. In our study, Gnathostomatomorpha forms a sister branch with Rhigonematoidea + Ascaridomorpha, which is consistent with previous studies^47,51,56^. However, previous studies have also suggested that Gnathostomatomorpha is nested within Ascaridomorpha^57^, or that Gnathostomatomorpha forms a sister branch with Ascaridomorpha + Oxyuridomorpha + Dracunculoidea + Spiruromorpha^58^.

The phylogenetic trees derived from two analytical methods exhibited a high degree of similarity in their topological structure. Notably, the branching patterns of the trees within the Gnathostomatomorpha clade displayed confuseing characteristics. Furthermore, dissimilar topologies were observed in the Oxyuridomorpha clade. Our results revealed that *W. compar* was sister to *W. siamensis*, with high statistical support (Bayesian posterior probability = 1.00; bootstrap support = 100). In Oxyuridomorpha, *W. compar* and *W. siamensis* are clustered together and have closer relationships with *S. obvelata* than with *E. vermicularis* in both trees, which is further supported by the comparison of gene orders and the genetic distance (PCGs) in this study. In previous research, it was observed that *A. tetraptera* and *S. obvelata* exhibited clustering, while *P. ambiguus* was identified as the sister species to *O. equi*^18^. Another study confirmed that *W. siamensis* was the sister species to *P. ambiguus*, as mentioned in a prior investigation^19^. Additionally, a recent study^47^ found *P. ambiuguus* to be sister to *A. tetraptera.* However, our results differ from these findings, possibly due to the limited availability of the complete mt genome of pinworms. In the present study, preference was given to BI tree classification owing to its higher support values. Despite pinworms are nematodes of human and animal health significance, the sequencing and publication of mitochondrial genomes have been limited to only seven species. Consequently, there is a need to increase taxon sampling in order to facilitate future phylogenetic investigations of this infraorder using mt genomic datasets.

The systematics of the suborder Spiruromorpha (Spiruromorpha, Oxyuridomorpha, Rhigonematoidea, Gnathostomatomorpha and Ascaridomorpha) has been controversial for decades. However, sequence analysis based on mt genomes presents roughly similar phylogenetic trees, which differ from those constructed based on 18S rRNA and single mitochondrial genes. To date, phylogenetic analysis based on 18S rRNA genes has accumulated more than 2700 sequences^59^, covering many but not all nematode taxa. However, it lacks the resolution needed to elucidate the phylogenetic relationships of the entire nematode tree, as different taxa exhibit different gene mutation rates, meaning that any single marker is unlikely to perform equally well in inferring the phylogeny of all nematode taxa. A better strategy for analysing nematode phylogeny involves targeting multiple genes rather than one gene. A large number of genes can help offset the adverse effects of recalcitrant genes in the analysis^60,61^. An analysis based on mt genomes fits this trend better, perhaps representing a better future for systematics.

## Conclusions

The present study identified the complete mt genome sequence of *W. compar*, enriching the mt genome database of Oxyuridae. This is the first complete mt genome isolated from the body of Chinese porcupines and belongs to the same *Wellcomia* genus as *W. siamensis* previously isolated from Korea. We have compared the results with the mt genome of other Oxyuridomorpha pinworms. The gene sequence arrangement results show that *W. compar* is more closely related to *W. siamensis* and *S.obvelata*. Based on the mt genome sequence, we have determined the phylogenetic relationships within Spirurina, and the phylogenetic analysis indicates that *W. compar* is the sister group of *W. siamensis*, which is consistent with the gene arrangement results. In the phylogenetic analysis, we found that the species support of Oxyuridomorpha was low and inconsistent. Therefore, we believe that it is necessary to further investigate additional taxonomic groups and sequencing data to understand and document the evolution of this important group. This mt genome provides a new genetic marker for studying the molecular epidemiology, population genetics, and systematics of parasitic pinworms in animals and humans, and is of great significance for the diagnosis, prevention, and control of parasitic diseases in porcupines and other animals.

### Supplementary Information

Supplementary Information 1.

Supplementary Information 2.

## Data Availability

All data supporting the conclusions of this article are included in this article. The data of *W. compar* in this study can be available from Genbank (MW059037).

## Abbreviations

mt: Mitochondrial
rRNA: Ribosomal RNA
tRNA: Transfer RNA
CDS: Coding sequences
PCG: Protein-coding gene
ML: Maximum likelihood
BI: Bayesian inference
NCR: Non-coding region
