# Supplementary material for: The complete mitochondrial genome of Wellcomia compar (Spirurina: Oxyuridae) and its genome characterization and phylogenetic analysis
Source: Sci Rep. 2023 Sep 2;13:14426. doi: 10.1038/s41598-023-41638-9 (PMC10475117; doi:10.1038/s41598-023-41638-9)
Supplement: Supplementary file 2 — Supplementary Information 2. [file 41598_2023_41638_MOESM2_ESM.docx]

**The complete mitochondrial genome of *Wellcomia compar* (Spirurina: Oxyuridae) and its genome characterization and phylogenetic analysis**

**Chunmao Huo^1,†^, Fengyun Bao^1,†^, Hong Long^1^, Tingyang Qin^1^, and Shibin Zhang^1,^***

^1^ Laboratory Animal Center of Zunyi Medical University, Zunyi 563006, China;

* Corresponding authors. Shibin Zhang; E-mail: [18300955528@139.com](mailto:18300955528@139.com)

† Chunmao Huo and Fengyun Bao equally to this work.

**Supplement material, Table S1**

List of Spirurina nematode species and outgroup used in this paper

| **Genbank** | **Species** | **Family** | **Length** | **AT%** |
| --- | --- | --- | --- | --- |
| GU070737 | *Trichuris suis* | Trichuridae | 14,436 | 71.5 |
| NC 018363 | *Thelazia callipaeda* | Thelaziidae | 13,668 | 74.6 |
| NC 021135 | *Spirocerca lupi* | Thelaziidae | 13,780 | 73.7 |
| MW971502 | *Pingus sinensis* | Quimperiidae | 13,874 | 68.7 |
| NC 016128 | *Cucullanus robustus* | Cucullanidae | 13,972 | 71.6 |
| NC 024020 | *Rhigonema thysanophora* | Rhigonematidae | 15,015 | 67.7 |
| MH931178 | *Physaloptera rara* | Physalopteridae | 13,735 | 72.4 |
| NC 016127 | *Heliconema longissimum* | Physalopteridae | 13,610 | 79.1 |
| EU281143 | *Enterobius vermicularis* | Oxyuridae | 14,010 | 71.2 |
| NC 027190 | *Oxyuris equi* | Oxyuridae | 13,641 | 67.8 |
| KT764937 | *Aspiculuris tetraptera* | Heteroxynematidae | 13,700 | 70.2 |
| KT900946 | *Syphacia obvelata* | Oxyuridae | 14,231 | 74.1 |
| NC 016129 | *Wellcomia siamensis* | Oxyuridae | 14,128 | 77.9 |
| NC 028345 | *Passalurus ambiguus* | Oxyuridae | 14,023 | 71.6 |
| NC 021643 | *Ascaridia columbae* | Ascaridiidae | 13,931 | 71.1 |
| NC 042411 | *Heterakis dispar* | Heterakidae | 13,995 | 69.8 |
| NC 062325 | *Tetrameres grusi* | Tetrameridae | 13,709 | 71.6 |
| NC 032073 | *Gnathostoma doloresi* | Gnathostomatidae | 13,809 | 70.5 |
| NC 027726 | *Gnathostoma spinigerum* | Gnathostomatidae | 14079 | 71.1 |
| NC 034239 | *Gnathostoma nipponicum* | Gnathostomatidae | 14093 | 74.5 |
| NC 080314 | *Gnathostoma binucleatum* | Gnathostomatidae | 14067 | 71.5 |
| HQ186250 | *Loa loa* | Onchocercidae | 13,590 | 75.6 |
| KT599912 | *Onchocerca volvulus* | Onchocercidae | 13,769 | 73.3 |
| NC 005305 | *Dirofilaria immitis* | Onchocercidae | 13,814 | 74.2 |
| NC 016197 | *Acanthocheilonema viteae* | Onchocercidae | 13,724 | 73.6 |
| NC 044071 | *Setaria labiatopapillosa* | Setariidae | 13,950 | 78.9 |
| NC 024931 | *Philometroides sanguineus* | Philometridae | 14,378 | 72.7 |
| MK820679 | *Anisakis simplex* | Anisakidae | 13,899 | 71.4 |
| NC 015927 | *Baylisascaris schroederi* | Ascarididae | 14,778 | 68.6 |
| NC 016198 | *Ascaris lumbricoides* | Ascarididae | 14,281 | 71.9 |
| NC 024037 | *Contracaecum osculatum* | Anisakidae | 13,823 | 70.2 |
| NC 024884 | *Parascaris univalens* | Ascarididae | 13,920 | 70.6 |
| NC 027163 | *Pseudoterranova azarasi* | Anisakidae | 13,954 | 70.7 |

**Supplement material, Table S2**

Best-fit partition model for BI

| Subset partitions | Best model |
| --- | --- |
| P1: (atp6 codon1, nad2 codon1, nad3 codon1, nad4L codon1, nad4 codon1, nad5 codon1, nad6 codon1) | GTR+F+I+G4 |
| P2: (atp6 codon2, nad6 codon2) | GTR+F+G4 |
| P3: (atp6 codon3, cox3 codon3, cytb codon3, nad4L codon3, nad6 codon3) | GTR+F+G4 |
| P4: (cox1 codon1) | GTR+F+G4 |
| P5: (cox1 codon2) | GTR+F+G4 |
| P6: (cox1 codon3, cox2 codon3, nad1 codon3, nad2 codon3, nad3 codon3, nad4 codon3, nad5 codon3) | GTR+F+G4 |
| P7: (cox2 codon1, cox3 codon1, cytb codon1, nad1 codon1) | GTR+F+I+G4 |
| P8: (cox2 codon2, cox3 codon2, cytb codon2, nad1 codon2, nad2 codon2, nad3 codon2, nad4L codon2, nad4 codon2, nad5 codon2) | GTR+F+I+G4 |

**Supplement material, Table S3**

Best-fit partition model for ML

| Subset partitions | Best model |
| --- | --- |
| P1: (atp6 codon1, nad2 codon1, nad3 codon1, nad4L codon1, nad4 codon1, nad5 codon1, nad6 codon1) | TN+F+I+I+R4 |
| P2: (atp6 codon2, nad6 codon2) | GTR+F+G4 |
| P3: (atp6 codon3, nad4L codon3, nad6 codon3) | TN+F+G4 |
| P4: (cox1 codon1, cox2 codon1) | TVM+F+I+G4 |
| P5: (cox1 codon2) | GTR+F+G4 |
| P6: (cox1 codon3, nad1 codon3, nad3 codon3, nad4 codon3, nad5 codon3) | TIM3+F+G4 |
| P7: (cox2 codon2, cox3 codon2, cytb codon2, nad1 codon2, nad2 codon2, nad3 codon2, nad4L codon2, nad4 codon2, nad5 codon2) | GTR+F+I+G4 |
| P8: (cox2 codon3, cox3 codon3, cytb codon3, nad2 codon3) | TIM3+F+G4 |
| P9: (cox3 codon1, cytb codon1, nad1 codon1) | TIM3+F+I+G4 |
